# How Does Radiation Affect Curcumin Raw Material?

**DOI:** 10.3390/ijms25052524

**Published:** 2024-02-21

**Authors:** Natalia Rosiak, Ewa Garbiec, Waldemar Bednarski, Robert Skibiński, Kornelia Lewandowska, Aleksandra Bazan-Woźniak, Robert Pietrzak, Judyta Cielecka-Piontek, Przemysław Zalewski

**Affiliations:** 1 Department of Pharmacognosy and Biomaterials, Poznan University of Medical Sciences, Rokietnicka 3, 60-806 Poznan, Poland; 2 Institute of Molecular Physics, Polish Academy of Sciences, Smoluchowskiego 17, 60-179 Poznan, Poland; 3 Department of Medicinal Chemistry, Medical University of Lublin, Jaczewskiego 4, 20-090 Lublin, Poland; 4 Faculty of Chemistry, Adam Mickiewicz University in Poznan, Uniwersytetu Poznańskiego 8, 61-614 Poznan, Poland

**Keywords:** curcumin, electron beam irradiation, antioxidant

## Abstract

Turmeric, known for its curcuminoid-rich rhizome, particularly curcumin, exhibits notable antioxidant and antiviral properties. The likelihood of microbial contamination necessitates finding reliable techniques for subjecting the sample to radiation from this plant-based raw material. One alternative is to expose curcumin to radiation (e-beam), which was carried out as part of this research. Confirmation of the lack of curcumin decomposition was carried out using HPLC-DAD/MS techniques. Additionally, using the EPR technique, the generated free radicals were defined as radiation effects. Using a number of methods to assess the ability to scavenge free radicals (DPPH, ABTS, CUPRAC, and FRAP), a slight decrease in the activity of curcumin raw material was determined. The analysis of the characteristic bands in the FT-IR spectra allowed us to indicate changes in the phenolic OH groups as an effect of the presence of radicals formed.

## 1. Introduction

Turmeric (*Curcuma longa* L.), a perennial herb indigenous to South Asia and classified under the *Zingiberaceae* family [1], is distinguished by its rhizome, which contains a considerable amount of curcuminoids. Among these curcuminoids, curcumin (CUR) stands out as the most abundant and significant compound [2,3], displaying notable antioxidant [4] and antiviral properties [5]. Because of its anti-inflammatory properties [6], CUR might mitigate the inflammatory processes implicated in conditions, such as arthritis [7]. It can also potentially protect against neurodegenerative conditions like Alzheimer’s disease and Parkinson’s disease. It possesses the ability to impede the aggregation of aberrant proteins within the brain and prevent nerve cell damage [8,9,10]. Considering the broad health-promoting properties of turmeric and the widespread use of this raw material for pharmaceutical applications and as a food ingredient, it is worth paying attention to its appropriate quality amid the globalization economy. According to a recent investigation conducted by Grand View Research, the estimated global curcumin market size was USD 58,199.4 thousand in 2020, with an anticipated increase to USD 65.36 million in 2021. Projections indicate a compound annual growth rate (CAGR) of 16.1% from 2020 to 2028, with an expected market value of USD 191.89 million by 2028. Notably, the pharmaceutical application held the predominant market share at 51.82% in 2020 [11].

However, a significant challenge associated with the utilization of CUR is the potential contamination of the plant source as a result of the development of selected bacteria and/or fungi. As a result, curcumin raw materials may be contaminated with aflatoxins [12,13]. They are a class of toxic compounds classified as mycotoxins, synthesized by specific strains of fungal molds, in particular, *Aspergillus flavus*. Aflatoxins exhibit significant carcinogenic properties, particularly concerning hepatocellular carcinoma, a form of liver cancer. Beyond their carcinogenic impact, aflatoxins can induce various health complications, including renal impairment, developmental abnormalities in juveniles, immunotoxicity, DNA harm, and hepatotoxic consequences [14].

Currently, the sole means of eliminating aflatoxins from raw plant material is to prevent their formation. One highly effective method employed for this purpose is sterilization through ionizing radiation, which eliminates microorganisms, such as bacteria, viruses, and fungi, by inducing DNA and vital cellular structure disruption. Sterilization through ionizing radiation utilizes sources, such as beta (β), gamma (γ) radiation, or high-energy electron beams (e-beam).

Γ-radiation is acknowledged in the scientific literature for its numerous benefits, including substantial penetration depth and a high level of sterility assurance [15], and its application for sterilizing turmeric is under discussion in the literature [16,17,18,19]. At a dosage of 5 kGy, irradiation has demonstrated an advantage in enhancing the extraction efficiency of CUR, attributed to the damage to the cell membrane caused by radiation exposure [16], and at a dosage of 10 kGy, γ-radiation exhibited no noticeable impact on the antioxidant activity of the tested turmeric extracts [20]. The safety of doses up to 10 kGy concerning the antioxidant effect of turmeric was subsequently affirmed by a later study [19].

Nevertheless, drawbacks such as a low dose rate, prolonged processing duration, and challenges associated with the cobalt isotope used as the radiation source, encompassing shortages in 60Co irradiation capacity, regulatory compliance, and associated costs, have prompted the exploration of alternative sterilization methods, with e-beam emerging as one of the alternatives [21]. E-beam irradiation is a cold method known for imparting a high degree of sterility assurance, accommodating high doses, and facilitating very short processing durations. However, it is essential to emphasize its characteristic of low penetration depth [15]. The impact of this form of radiation has been previously investigated in numerous raw plant materials, including almonds [22,23], blueberries [24], broccoli [25], grapefruit and lemons [26], ginseng [27], and soybean [28].

To date, a single study has employed e-beam technology for sterilizing turmeric, utilizing doses spanning from 5 to 30 kGy [29]. The authors investigated the impact of these doses on the elimination of specific bacteria, the extraction of turmeric components, and the radiolysis of curcuminoids utilizing the high-performance liquid chromatography (HPLC) method. In both pre- and post-irradiation samples of turmeric powder extract, ferulic acid, vanillin, and 4-vinylguaiacol were identified among the curcumin chain-cleavage products. Following irradiation with a dose of 30 kGy, only the content of 4-vinylguaiacol showed an increase, amounting to 15%. While ionizing radiation is advantageous for products sensitive to heat, leaving no chemical residues, it is crucial to note the tendency, recognized so far for various synthetic compounds, to undergo chemical instability when exposed to ionizing radiation [30,31,32,33,34,35,36,37]. The probability of chemical instability increases with a rise in the radiation dose, and it is important to consider that, concerning the elimination of fungi like *Aspergillus flavus*, the literature indicates that a considerably high dose, approximately 10 kGy, is adequate for complete eradication. This is exemplified in the case of edible split beans from the wild legume *Canavalia maritima* [38]. Additionally, a dose of 20 kGy has been demonstrated as effective in reducing aflatoxins produced by *Aspergillus flavus* in maize slurry [39].

Therefore, it is essential to assess the impact of radiation sterilization on the durability and antioxidative characteristics of the most important turmeric active ingredient, CUR. This evaluation requires the application of methodologies not employed in studies carried out thus far for CUR, specifically using the electron paramagnetic resonance (EPR) method to observe and analyze the presence and behavior of free radicals produced during the process and FT-IR spectroscopy to assess molecular alterations of the molecule.

The study aimed to assess the sensitivity of CUR (from *Curcuma longa*, assay ≥ 65% of curcuminoids) to a radiation sterilization dose of 25 kGy, following EN 522 standards, utilizing EPR, FT-IR, HPLC-DAD, and HPLC-MS methods, and to analyze the consequent impact on its antioxidant properties.

## 2. Results and Discussion

The assessment of the CUR stability against radiation after exposure to electron beam irradiation at doses of 25 kGy was performed at two different time points, namely, 49 h and 595 h, using techniques including FT-IR, HPLC-DAD, and HPLC-MS. To determine whether irradiation had an impact on the antioxidant characteristics, DPPH, ABTS, CUPRAC, and FRAP assays were conducted. The main objective of this research was to determine whether exposure to ionizing radiation brings modifications in the chemical composition of the CUR and its antioxidative properties.

### 2.1. Electron Paramagnetic Resonance

After exposure to ionizing radiation, additional free radicals, which are highly reactive species, can be formed. The EPR technique was used to monitor the decay of free radicals over time.

**Figure 1:**
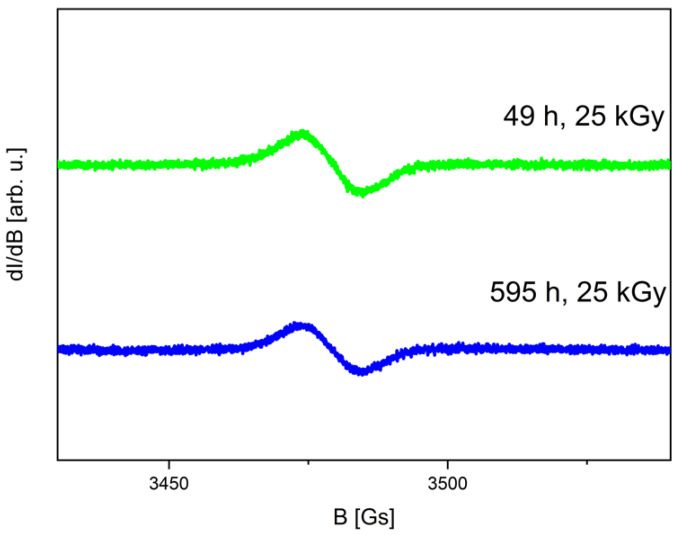
EPR spectra of irradiated CUR recorded 49 h (green) and 595 h (blue) after 25 kGy dose irradiation.

The EPR spectra of CUR, recorded at 49 and 595 h after exposure to radiation with a dose of 25 kGy, are depicted in Figure 1.

**Figure 2:**
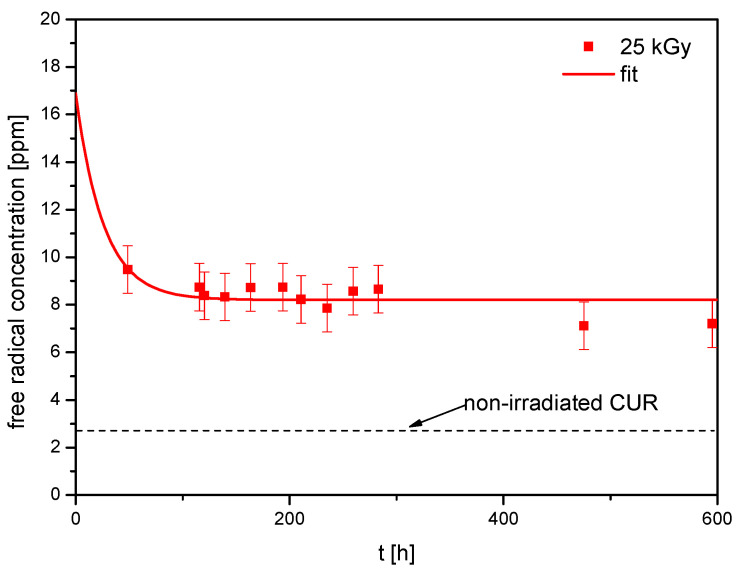
The concentration of free radical vs. time after irradiation (25 kGy) and for the non-irradiated sample marked by a dashed line.

The EPR spectrum exhibits a single line characterized by a linewidth ΔBpp of 10.8 ± 0.5 Gs and the spectroscopic splitting factor g of 2.0040 ± 0.0005. It can be seen that the intensity of the spectrum for the irradiated CUR decreases with time, indicating the presence of unstable forms of free radicals. The variation in free radical concentration over time following irradiation, determined from EPR spectra, is illustrated in Figure 2.

The equation provided can be used to explain how the concentration of free radicals decreases over time in the irradiated sample [40,41]:

$$C_{tot}\left(t\right)=C_{s}+C_{u}e^{-\frac{t}{T}}$$

where *C_tot_*(*t*) represents the overall concentration of free radicals assessed at a specific moment *t* following radiation exposure. *C_s_* denotes the concentration of stable radicals, *C_u_* signifies the concentration of unstable free radicals, *t* denotes the time elapsed post-radiation, and *T* signifies the average lifespan of unstable radicals.

The data calculated from the analysis for the irradiated sample are as follows: *C_tot_* (*t =* 0) = 16.91 ± 4.18 ppm, *C_s_* = 8.21 ± 0.18 ppm, *C_u_* = 8.7 ± 4.0 ppm. The average lifetime of unstable radicals is 26 ± 10 h. The concentration of stable radicals *C_s_* for the non-irradiated CUR reaches 2.7 ± 0.4 ppm, so it is about three times lower than for the irradiated sample.

Notwithstanding its many advantages, one of the limitations associated with the use of radiation sterilization is the possibility of inducing physical and/or chemical disturbances in the sterilized substance. The unavailability of a well-defined hyperfine structure hinders the precise localization of bond breakages in CUR molecules through EPR analysis.

Radiation exposure frequently results in the generation of free radicals. Notably, CUR’s antioxidant activity is predicated on its capacity to scavenge free radicals. Following irradiation of CUR with a dose of 25 kGy, the total concentration of free radicals was 16.91 ± 4.18 ppm. Comparative analysis with existing literature reveals that CUR exhibits lower resistance to radical damage induced by irradiation when compared with other phytochemicals of plant origin, namely, resveratrol, rutin, and quercetin. For these compounds, the total radical concentrations after irradiation with an equivalent dose were 0.24 ppm, 2.42 ppm, and 0.42 ppm, respectively [40,41].

The levels of irradiation-induced free radicals in the sample, as evidenced by EPR, decrease over time. The plateau is established at a level approximately three times higher than that observed in the unirradiated sample, signifying the stability of certain radicals formed during irradiation. The concentrations of stable or long-lived radicals that we measure after irradiation are close to the concentrations occurring naturally in many plants, even those in which there was no oxidative stress or irradiation [42]; therefore, their significant impact on human health is not expected.

### 2.2. Fourier Transform Infrared Spectroscopy

**Figure 3:**
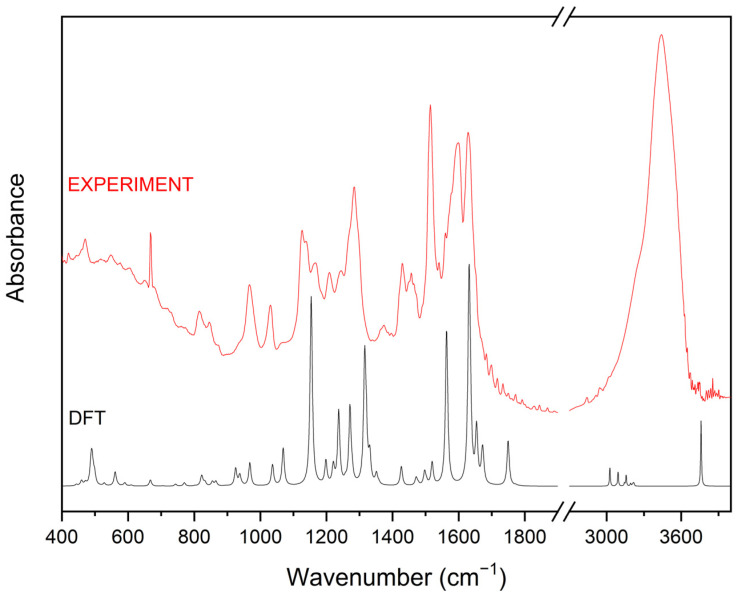
Calculation (black-DFT) and experimental (red) IR absorption spectra of CUR at room temperature.

To assess the alterations following the exposure of CUR to irradiation, FT-IR spectroscopy was employed. Theoretical and experimental absorption spectra in the IR range of CUR are depicted in Figure 3.

The experimental IR absorption spectra of CUR are different from the calculation DFT spectrum. The calculation spectrum has more but narrower bands than the experimental IR spectrum. That is why one broad band observed in the experimental spectrum has more components and is related to larger amounts and types of vibration. The comparison of the frequencies calculated by the DFT–B3LYP method with the experimental values reveals the overestimation of the calculated vibrational modes due to neglect of anharmonicity in the real system.

The experimental FT-IR spectrum shows that CUR exists only in the enol form [43]. The absorption spectrum of CUR is dominated by eight strong bands at 967, 1030, 1126, 1283, 1514, 1597, 1628, and 3440 cm^−1^ (Table S1). The first two bands are mainly related to the deformation of the molecule and the wagging vibration of the C-H bonds, respectively. Whereas the next three bands are associated with stretching vibration of the C-C and C-O bonds. The bands at 1597 and 1628 cm^−1^ correspond to the stretching vibration of the C=C and C=O bonds. The strongest band at 3440 cm^−1^ is related to the stretching vibration of the O-H bonds, but it has also an additional component associated with the stretching vibration of the C-H bonds. For CUR, there are also characteristic bands associated with the bending vibration of the C-O-H bond; they are located for example at 1208, 1243, 1373, and 1560 cm^−1^. Less intense bands below 900 cm^−1^ and in the range 1400–1500 cm^−1^ are mainly related to the wagging and rocking vibration of the C-H bonds.

**Figure 4:**
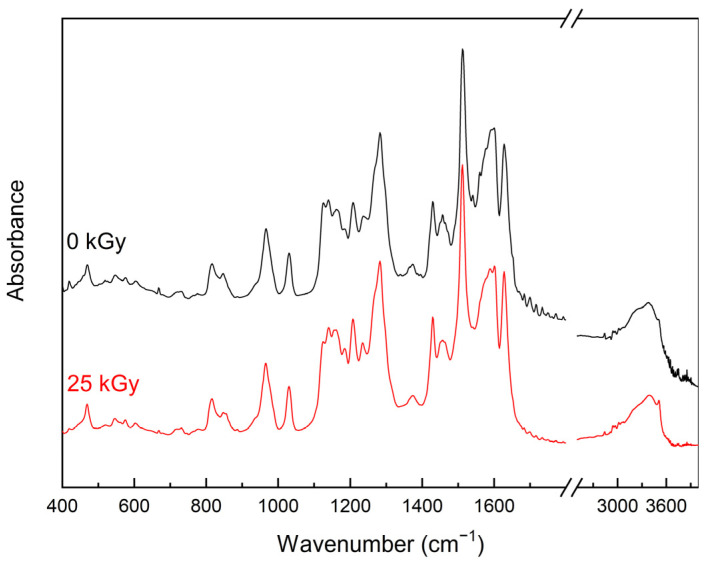
FT-IR spectra of unirradiated and irradiated CUR.

In the absorption spectrum for the CUR after irradiation, all bands recorded before the process are observed, but the relative intensities of their maxima change (Figure 4).

The differences are visible in the bands associated with the stretching vibration of the C-O (1243, 1283, 1514 cm^−1^), C=O (1628 cm^−1^), and C=C (1597 cm^−1^) bonds and wagging, rocking vibration of the C-H (1166, 1429, 1457 and 1465 cm^−1^) bonds, and bending vibration of the C-O-H (1208, 1373 cm^−1^) bonds. Observed changes may suggest that irradiation induced the free radicals formation of CUR, which was also confirmed by the EPR study.

### 2.3. HPLC-DAD and HPLC-MS Analysis

To assess the chemical stability of the samples following exposure to a radiation dose of 25 kGy, HPLC-DAD/MS methods were used. After carrying out a series of measurements, chromatograms for bisdemethoxycurcumin, demethoxycurcumin, and CUR were obtained (Figure S1). The presence of the same compounds was confirmed by HPLC-MS (Table 1).

The presence of these compounds is attributed to the composition of the examined sample, which exhibited a curcuminoid content of ≥65%. Curcuminoids comprise a blend of CUR, along with its two derivatives, demethoxycurcumin and bisdemethoxycurcumin [44]. It should be noted that these compounds are similarly detected in non-irradiated samples. Both methods did not indicate the existence of additional compounds, thereby precluding the observation of degradation products in the presence of ionizing radiation.

### 2.4. Antioxidant Properties

**Figure 5:**
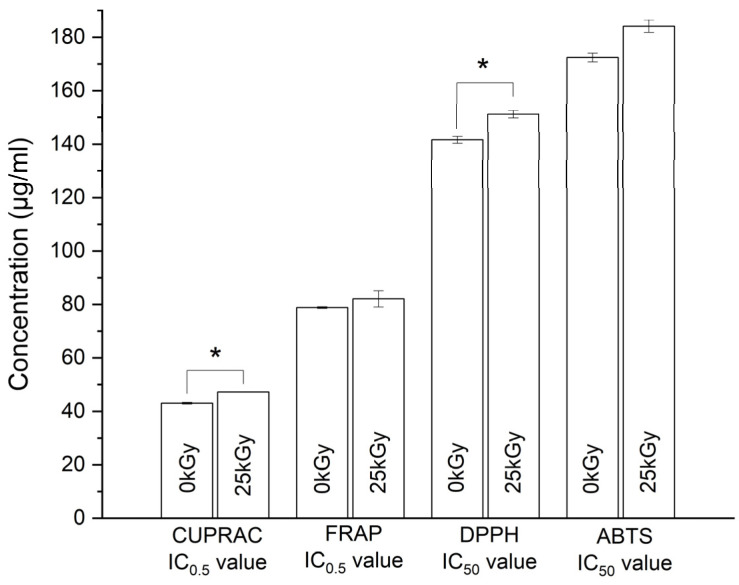
Summary of antioxidant test results. *X*-axis: IC_50_ values for DPPH and ABTS assays, or IC_0.5_ values for CUPRAC and FRAP assays. *Y*-axis: Concentration of the sample; 0 kGy—non-irradiated CUR; 25 kGy—irradiated CUR. Asterisk (*) indicates significance with *p* ≤ 0.05.

To investigate the antioxidant properties of a substance comprehensively, relying on a single test is inadequate. Therefore, to examine how irradiation influenced the antioxidant capabilities of CUR, various methods were employed. These methods involved assessing the impact of antioxidants on the reduction of metal ions, specifically iron (FRAP) and copper (CUPRAC), as well as evaluating their capacity to scavenge synthetic radicals (DPPH and ABTS). The bar graphs in Figure 5 depict the IC_50_ values for the DPPH and ABTS assays, along with the IC_0.5_ values for the CUPRAC and FRAP assays.

The heightened antioxidant activity is reflected in the lower IC_50_ and IC_0.5_ values. Both pre- and post-irradiation, CUR displayed antioxidant activity across conducted tests. In tests involving model radicals, the IC_50_ value in the DPPH assay was 141.62 ± 1.31 µg·mL^−1^ for non-irradiated CUR and 151.13 ± 1.40 µg·mL^−1^ after irradiation. In the test involving ABTS radical, the IC_50_ values were 172.41 ± 1.63 µg·mL^−1^ and 184.13 ± 2.33 µg·mL^−1^ before and after irradiation, respectively. This implies a lower concentration of CUR is needed to neutralize 50% of the initial DPPH radical compared to ABTS. The higher reactivity of the ABTS radical compared to the DPPH radical [4] could account for this difference.

Despite varied reports on the mechanism of CUR’s antioxidant activity [45,46,47,48], a general consensus suggests dependence on factors such as the reaction environment (solvent and pH) and the nature of free radicals. These factors dictate which mechanism dominates CUR’s antioxidant activity. In contrast to DPPH radical reactions involving H atom transfer, reactions with ABTS radicals involve a process of transferring electrons. The likely reaction site for both mechanisms is the phenolic part of CUR. In our study, FT-IR analysis revealed changes in the phenolic OH groups in samples following irradiation. These changes are likely a result of oxidative damage induced by the presence of free radicals. This may elucidate the observed decrease in antioxidant activity after irradiation. A parallel decrease in antioxidant activity in the ABTS radical assay was noted in the study by Rosiak et al. [40] for resveratrol irradiated with a dose of 25 kGy. Another plausible explanation is the presence of free radicals in the sample [18], as confirmed by EPR studies.

The results from CUPRAC and FRAP tests reveal that CUR demonstrates the capacity to chelate metal ions, specifically copper and iron. This outcome aligns with existing literature, suggesting that compounds featuring C-OH and C=O functional groups tend to exhibit metal-chelating properties. In the case of CUR, the presence of hydroxyl and methoxyl groups arranged favorably has been proposed as contributing to its ability to form chelates with metal ions. Ak et al. [4] have identified the phenolic rings and groups in the heptadienone link of CUR as active sites within the molecule.

Slightly reduced CUR activity after irradiation was observed in both tests, as indicated by the results: 43.04 ± 0.31 µg·mL^−1^ and 47.24 ± 0.10 µg·mL^−1^ before and after exposure, respectively, in the CUPRAC assay and 78.83 ± 0.30 µg·mL^−1^ and 82.08 ± 3.05 µg·mL^−1^ before and after exposure, respectively, for the FRAP test. An explanation may be derived from the observed changes in the FT-IR spectrum, suggesting that alterations in the chemical structure, particularly in the identified active groups, could be a contributing factor to the slight decrease in CUR activity post-exposure, similar to what was observed in the DPPH and ABTS assays.

Our research indicates that, despite maintaining chemical stability, irradiation induces observable changes in the CUR molecule, influencing its antioxidant characteristics. The detected variations in antioxidant activity, although subtle, demonstrated statistical significance in both the DPPH and CUPRAC assays. Therefore, these alterations should be acknowledged as potential consequences of irradiation. Furthermore, it is crucial to recognize that CUR’s efficacy extends beyond its anti-free radical attributes to include anti-inflammatory properties [6] and enzyme modulation [49]. Consequently, further investigations are warranted, employing diverse mechanisms and techniques to assess antioxidant activity. Additionally, in vivo models should be explored to ascertain the broader implications of post-irradiation alterations on antioxidant properties and other facets of CUR’s actions not addressed in this study, such as enzyme inhibition and anti-inflammatory effects.

Future research should also explore the stability and long-term changes, acknowledging that while no additional effects of irradiation in the mechanism of damage caused by free radicals formed are anticipated, long-term changes in antioxidant activity cannot be excluded.

## 3. Materials and Methods

### 3.1. Standards and Reagents

The curcumin sample (derived from *Curcuma longa*, assayed for a curcuminoid content of ≥65%), the reference standard of curcumin (purity > 99.5%), potassium bromide, 2,2-diphenyl-1-picrylhydrazyl, iron (III) chloride hexahydrate, 2,4,6-Tri(2-pyridyl)-s-triazine, neocuproine, potassium persulfate, and 2,2′-Azino-bis(3-ethylbenzothiazoline-6-sulfonic acid) were acquired from Merck (Warsaw, Poland). An analytical amount of ammonium acetate, weighed, along with hydrochloric acid 0.1 N and methanol, was supplied by Chempur (Piekary Śląskie, Poland). Copper (II) chloride dihydrate, acetic acid (99.5%), ethanol (96%), sodium acetate trihydrate, glacial acetic acid, and formic acid were supplied by Avantor Performance Materials Poland S.A. (Gliwice, Poland). Acetonitrile of an HPLC grade was supplied by Romil (Waterbeach, Cambridgeshire, UK). High-quality pure water was prepared using a Direct-Q 3 UV purification system (Millipore, Molsheim, France, model Exil SA 67120).

### 3.2. Irradiation

Five milligrams of CUR samples was measured and placed in colorless glass vials, sealed with plastic stoppers. The vials containing CUR samples were exposed to a 9.96 MeV electron beam with a current intensity of 6.2 μA using a linear electron accelerator LAE 13/9. The irradiation was carried out at a dose of 25 kGy, and the electron energy was 10 MeV with an average beam current of 6.2 μA. The nominal doses were determined by a calorimeter, assumed to be accurate within ±0.372%. The electron energy was measured using an aluminum wedge. The irradiation took place at room temperature on a conveyor, lasting for 2 s. The dose distribution on the conveyor was homogeneous within the confidence interval of ±10%. Following irradiation, the glass of the jar underwent a color change from colorless to brown.

### 3.3. Electron Paramagnetic Resonance (EPR) Spectroscopy

The Bruker ELEXSYS 500 X-band (9.4 GHz) spectrometer (Bruker, Billerica, MA, USA) was applied to detect the presence of free radicals and quantify their concentration. Samples of CUR in a solid state were positioned in quartz capillaries (Wilmad, Merc, Darmstadt, Germany), and then, they were put into the resonator. EPR experiments were carried out at room temperature, using a low microwave power (2 mW) to prevent EPR line saturation. The determination of the concentration of free radicals was performed by applying the methodology outlined by Mai et al. [42].

### 3.4. Fourier Transform Infrared (FT-IR) Spectroscopy

The FT-IR spectra were obtained using an IR Affinity-1 spectrometer (Shimadzu, Kyoto, Japan). For sample preparation, 1 mg of CUR was mixed with 300 mg of potassium bromide as a matrix material. Pellets with a diameter of 13 mm were created under a compaction pressure of 1.5 MPa. Absorption spectra were recorded in the wavenumber range of 4000–400 cm^−1^ with a resolution of 2 cm^−1^ (30 scans per spectrum).

### 3.5. High-Performance Liquid Chromatography (HPLC-DAD) Analysis

The assessment was conducted following a previously described method [50], utilizing the Dionex Ultimate 3000 analytical system (Dionex, Sunnyvale, CA, USA) and employing a Luna C18 column (Phenomenex, Warsaw, Poland) with a particle size of 5 µm and dimensions of 250 mm × 4 mm. The investigation utilized 5 mg samples of CUR sourced from *Curcuma longa*, with an assay confirming a curcuminoid content of ≥65%, both prior to and following exposure to irradiation. The samples were dissolved in a 100 mL mobile phase. The elution process employed a constant mobile phase composition of 1% acetic acid–acetonitrile in a ratio of 45:50 *v*/*v*. The flow rate of the mobile phase was consistently set at 1.0 mL·min^−1^ throughout the analysis. The injection volume was 20 µL. The test was conducted at a wavelength of 421 nm using the diode array detector (DAD) and at a temperature of 30 °C. The limits of detection (LODs) and quantifications (LOQs) for the curcumin, demethoxycurcumin, and bisdemethoxycurcumin are provided in Table S2 (Supplementary Materials).

### 3.6. HPLC-MS Analysis

The mass spectrometry analysis was conducted using the Agilent Accurate-Mass Q-TOF LC/MS G6520B system with a DESI ion source and an Infinity 1290 ultra-high-pressure liquid chromatography system consisting of a G4220A binary pump, a G4226A autosampler, a G1330B FC/ALS thermostat, a G4212A DAD, and a G1316C TCC module (Agilent Technologies, Santa Clara, CA, USA). The MassHunter workstation software B.04.00 was employed for system control, data acquisition, and qualitative analysis.

The separation of degradation products took place on a Hibar RP-18e 50 mm × 2.1 mm column with 2 µm particle size (Merck, Darmstadt, Germany). The mobile phase, composed of acetonitrile (A) and 0.1% of acetic acid (B), was used. The gradient elution was carried out at a constant flow of 0.3 mL·min^−1^ from 10% A (90% B) 0–0.5 min and then 10% A to 80% A (0.5–9 min) and the next 2 min post-time was performed to return to the initial condition.

The Q-TOF detector was tuned in the negative polarity—4 GHz, and key parameters were optimized based on a CUR analytical standard and set as follows: nebulizer pressure 40 psi, drying gas 10 L·min^−1^, gas temp. 300 °C, fragmentor voltage 200 V, capillary voltage 3500 V, skimmer voltage 65 V, and octopole 1 radio frequency voltage 750 V. The auto MS/MS mode with a mass range of 50–1050 *m*/*z* and an acquisition rate of 1.2 spectra/s (for MS and MS/MS data) was utilized for data collection.

Collision energy calculation employed the formula 2 V (slope) × (*m*/*z*)/100 + 6 V (offset), and a maximum of two precursors per cycle were selected with an active exclusion mode after 1 spectrum for 0.2 min. Measurement accuracy was ensured through reference mass correction and the selection of lock mass values at 112.9856 and 1033.9881 *m*/*z*.

### 3.7. Antioxidant Activity Study

Antioxidant assessment was conducted through the application of various assays, including DPPH, ABTS, CUPRAC, and FRAP.

The assessment of antioxidant activity against the DPPH radical was conducted following a previously described method [51], with modifications. In this procedure 25 μL of CUR samples derived from Curcuma longa, assayed for a curcuminoid content of ≥65%, both before and after irradiation dissolved in methanol at varying concentrations (0.05–0.30 mg·mL^−1^) was combined with 175 μL of 0.2 mM DPPH solution and incubated for 30 min at room temperature in the dark with agitation. Absorbance readings were taken at 517 nm using a UV/V is microplate spectrophotometer (Multiskan GO, Thermo Fisher Scientific, Waltham, MA, USA). The control consisted of 25 μL of methanol mixed with 175 μL of the DPPH solution.

For the ABTS assay, in accordance with the previously reported procedure [52], a quantity of 200 µL of the ABTS solution (prepared by dissolving 0.0384 g of ABTS in 10 mL of aqueous 2.45 mM potassium persulfate solution, allowing it to stand for 24 h, and subsequently diluting it with deionized water until the absorbance reached approximately 0.77 at 734 nm) was introduced to 50 µL of the CUR samples derived from *Curcuma longa*, assayed for a curcuminoid content of ≥65%, both before and after irradiation dissolved in methanol at varying concentrations (0.075–0.300 mg·mL^−1^). The mixture was then incubated in the dark for 10 min at room temperature. Following incubation, the absorbance was measured at λ = 734 nm using the same mentioned device.

The antioxidant capacity of the samples was assessed using the CUPRAC assay, following a method outlined previously [53]. In this procedure, 50 μL of CUR samples derived from *Curcuma longa*, assayed for a curcuminoid content of ≥65%, both before and after irradiation dissolved in methanol at varying concentrations (0.012–0.150 mg·mL^−1^) was mixed with 150 μL of CUPRAC reagent, which consists of equal parts of 7.5 mM ethanolic 96% neocuproine solution, 10 mM CuCl_2_·H_2_O solution, and ammonium acetate buffer with a pH of 7.0. Subsequently, the sample’s absorbance was measured at 450 nm (Multiskan GO, Thermo Fisher Scientific, Waltham, MA, USA) after a 30 min incubation in the dark at room temperature.

The FRAP assay, as previously described [54], utilized stock solutions of FRAP reagent comprising 300 mM acetate buffer (pH 3.6), 10 mM TPTZ solution in 40 mM HCl, and 20 mM FeCl_3_∙6H_2_O solution. The operational FRAP solution was created by combining 25.0 mL of acetate buffer, 2.5 mL of TPTZ solution, and 2.5 mL of FeCl_3_∙6H_2_O solution, followed by warming at 37 °C before application. In summary, 25.0 μL of CUR samples derived from *Curcuma longa*, assayed for a curcuminoid content of ≥65%, both before and after irradiation dissolved in methanol at varying concentrations (0.012–0.150 mg·mL^−1^), was mixed with 175.0 μL of FRAP solution, agitated, and incubated at 37 °C for 30 min in darkness. Subsequently, the absorbance was measured at 593 nm using the same device as mentioned.

The sample’s radical scavenging activity in the DPPH and ABTS assays was determined using the formula:

$$\mathrm{The}\;\mathrm{degree}\;\mathrm{of}\;\mathrm{radical}\;\mathrm{scavenging}\;(\%)=\frac{A_{0}-A_{i}}{A_{0}}\times 100\%$$

where $A_{0}$ is the absorbance of the control and $A_{i}$ is the absorbance of the sample.

The outcomes of the DPPH and ABTS assays are displayed as a plot depicting the percentage of inhibition against concentration. Conversely, the results of the CUPRAC and FRAP assays are represented in a plot showcasing absorbance versus concentration.

The IC_50_ or IC_0.5_ value was established through regression analysis, employing either linear or polynomial models.

y=ax+b

where *x* denotes the final sample concentrations, *y* signifies the inhibition ratios, and *a* and *b* denote the coefficients.

$$y=ax^{2}+bx+c$$

where *x* denotes the final sample concentrations, *y* signifies the inhibition ratios, and *a*, *b* as well as *c* stand for the coefficients.

The determination of the final sample concentration for IC_50_ involved substituting *Y* in the regression equation with 50. Similarly, for IC_0.5_, the substitution was made with 0.5. Antioxidant method performance parameters—limits of detection (LOD) and quantification (LOQ)—are provided in Table S3 (Supplementary Materials).

### 3.8. Statistical Analysis

Statistical analyses were performed utilizing Statistica 13.3 software (StatSoft, Krakow, Poland). The collected data underwent a one-way analysis of variance (ANOVA) followed by Duncan’s post hoc test. Significance was established at a probability level of *p* < 0.05. Results are presented as mean ± standard deviations.

## 4. Conclusions

Our experimental results reveal the resilience of curcuminoids, particularly curcumin, in response to electron beam (e-beam) radiation exposure at a dose of 25 kGy. Analytical techniques, including HPLC-DAD/MS, conclusively demonstrated the absence of curcumin degradation under these irradiation conditions.

Molecular changes in FT-IR spectra provided valuable insights into the specific modifications occurring in the phenolic OH groups of curcumin. These changes were attributed to the presence of radiation-induced free radicals, whose identification was accomplished with the EPR technique. These molecular changes were implicated in the subtle shifts observed in curcumin’s antioxidant activity, as demonstrated through assessments using research methodologies like DPPH, ABTS, CUPRAC, and FRAP, while maintaining the chemical stability of curcumin.

Our results underscore the complexity of the impact of radiation on the functional properties of curcumin and suggest a need for further exploration utilizing advanced models that can offer a more intricate understanding of changes in antioxidant activity induced by ionizing radiation. Such endeavors will be crucial for optimizing the application of radiation in preserving the quality and functionality of curcumin for diverse industrial uses, paving the way for advancements in the pharmaceutical and food industries.

## Figures and Tables

**Table 1:** HPLC/MS analysis results for irradiated sample.

Name	Retention Time (min)	Measured Mass (*m*/*z*)	Theoretical Mass (*m*/*z*)	Mass Error (ppm)	Molecular Ion Formula [M-H^−^]	MS/MS Fragmentation Ions (*m*/*z*)	MS/MS Fragment Formula
Curcumin 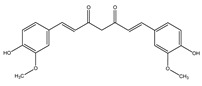	6.9	367.1167	367.1187	5.38	C_21_H_19_O_6_	217.0505 173.0603 158.0371 149.0604 132.0237	C_12_H_9_O_4_ C_11_H_8_O_2_ C_10_H_6_O_2_ C_9_H_9_O_2_ C_8_H_6_O_2_
Demethoxycurcumin 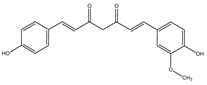	6.75	337.1084	337.1081	0.3	C_20_H_17_O_5_	217.0508 173.0609 158.0356 134.0219 119.0501	C_12_H_9_O_4_ C_11_H_8_O_2_ C_10_H_6_O_2_ C_8_H_4_O_2_ C_8_H_7_O
Bisdemethoxycurcumin 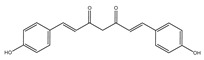	6.6	307.0967	307.0976	2.77	C_19_H_15_O_4_	187.0379 143.0476 158.0356 119.0491	C_11_H_9_O_3_ C_10_H_7_O C_10_H_6_O_2_ C_8_H_7_O

## Data Availability

Data are contained within the article and Supplementary Materials.

## Supplementary Materials

The supporting information can be downloaded at https://www.mdpi.com/article/10.3390/ijms25052524/s1.
