# Supplementary material for: How Does Radiation Affect Curcumin Raw Material?
Source: Int J Mol Sci. 2024 Feb 21;25(5):2524. doi: 10.3390/ijms25052524 (PMC10931914; doi:10.3390/ijms25052524)
Supplement: Supplementary file 1 [file ijms-25-02524-s001.zip › ijms-2865975-supplementary-done.pdf]

International Journal of *Molecular Sciences*

Supplementary materials

How does radiation affect curcumin raw material?

Natalia Rosiak[1], Ewa Garbiec[1], Waldemar Bednarski[2], Robert Skibiński[3], Kornelia Lewandowska[2], Aleksandra Bazan-Woźniak[4], Robert Pietrzak[4], Judyta Cielecka-Piontek[1,*], Przemysław Zalewski[1]

1 Department of Pharmacognosy and Biomaterials, Poznan University of Medical Sciences, Rokietnicka 3, 60-806 Poznan, Poland
2 Institute of Molecular Physics, Polish Academy of Sciences, Smoluchowskiego 17, 60-179, Poznan, Poland
3 Department of Medicinal Chemistry, Medical University of Lublin, Jaczewskiego 4, 20-090 Lublin, Poland
4 Faculty of Chemistry, Adam Mickiewicz University in Poznan, Uniwersytetu Poznańskiego 8, 61-614 Poznan, Poland
* Correspondence: jpiontek@ump.edu.pl

Table S1. Selected characteristic vibronic features of curcumin theory with application of 6-31G(d,p) basis and experiment bands of curcumin. s-stretching, b-bending, w-wagging, r-rocking, t-twisting, oop-outside of the plane, def. - deformation.

Calculation (cm^{-1})	Experimental (cm^{-1})	Band assignment
561	470	Def. all molecule
582	548	Def. all molecule
823	815	Def. benzene rings
855	846	C-H w
865	872	C-H w
968	967	Def. all molecule
1038	1030	C-H w
1069	1064	CH_2 t + C-O s + def. benzene rings
1154	1126	C-C s
1198	1166	C-H w
1238	1208	C-O-H b + C-H r
1270	1243	C-O s + C-O-H b + C-H r + CH_2 t
1320	1283	C-O s + C-H r
1426	1373	C-O-H b + C-C s in benzene rings
1470	1429	C-H r
1502	1457	C-H w in CH_3
1519	1465	C-H r in CH_3
1564	1514	C-O s + C-C s
1635	1560	C-C s + C-O-H b + C-H r
1655	1577	C=C s + C-C s + C-O-H b
1674	1597	C=C s
1749	1628	C=O s + C=C s
3025	2841	C-H s in CH_3
3156	2941	C-H s
3760	3440	O-H s

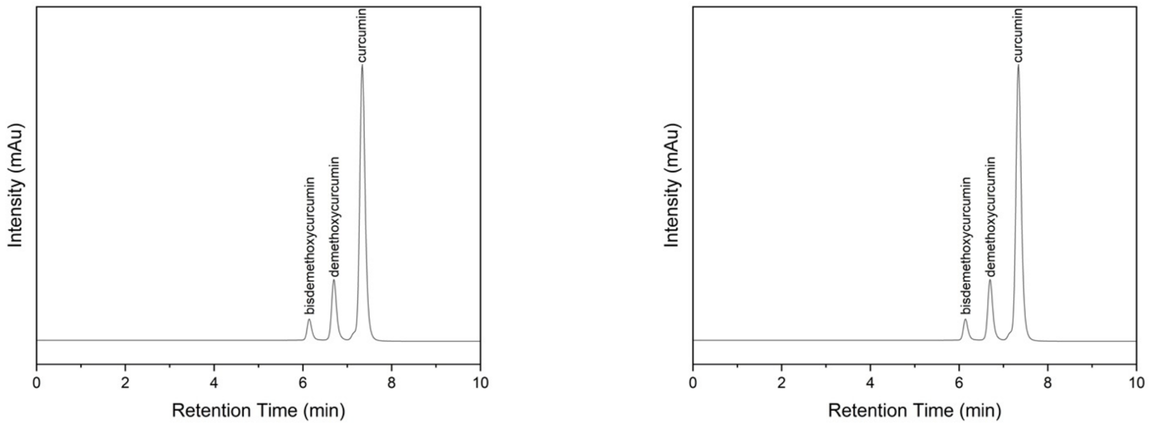

a b

Figure S1. The HPLC-DAD analysis of non-irradiated (a) and irradiated (b) sample (assay ≥65% of curcuminoids).

Table S2. Validation parameters of HPLC-DAD method.

Curcumin	
Parameter	Curcumin dissolved in mobile phase
Injection volume	20 µl
Linearity range (mg/mL)	0.0001 – 0.2
Correlation coefficient (r)	0.9999
a ± Sa	164512465 ± 1458461
b ± Sb	insignificant (α=0.05)
LOD (mg/mL)	0.0025
LOQ (mg/mL)	0.0076
Demethoxycurcumin	
Parameter	Demethoxycurcumin dissolved in mobile phase
Injection volume	20 µl
Linearity range (mg/mL)	0.0001 – 0.2
Correlation coefficient (r)	0.9998
a ± Sa	142586247 ± 1346285
b ± Sb	insignificant (α=0.05)
LOD (mg/mL)	0.0027
LOQ (mg/mL)	0.0081
Bisdemethoxycurcumin	
Parameter	Bisdemethoxycurcumin dissolved in mobile phase
Injection volume	20 µl
Linearity range (mg/mL)	0.0001 – 0.2
Correlation coefficient (r)	0.9997
a ± Sa	172157465 ± 1254585
b ± Sb	insignificant (α=0.05)
LOD (mg/mL)	0.0023
LOQ (mg/mL)	0.0074

Table S3. Antioxidant method performance parameters - limits of detection (LOD) and quantification (LOQ).

Assay	LOD (mg/mL)	LOQ (mg/mL)
ABTS 0 kGy	0.0031	0.0093
ABTS 25 kGy	0.0030	0.0090
CUPRAC 0 kGy	0.0009	0.0027
CUPRAC 25 kGy	0.0002	0.0007
DPPH 0 kGy	0.0031	0.0095
DPPH 25 kGy	0.0043	0.0131
FRAP 0 kGy	0.0004	0.0011
FRAP 0 kGy	0.0003	0.0008

0 kGy - non-irradiated CUR; 25 kGy - irradiated CUR
